# Accelerated 3D whole-heart non-contrast-enhanced mDIXON coronary MR angiography using deep learning-constrained compressed sensing reconstruction

**DOI:** 10.1186/s13244-024-01797-3

**Published:** 2024-09-19

**Authors:** Xi Wu, Xun Yue, Pengfei Peng, Xianzheng Tan, Feng Huang, Lei Cai, Lei Li, Shuai He, Xiaoyong Zhang, Peng Liu, Jiayu Sun

**Affiliations:** 1 Department of Radiology, Hunan Provincial People’s Hospital, The First Affiliated Hospital of Hunan Normal University, Changsha, China; 2 Department of Radiology, West China Hospital of Sichuan University, Chengdu, China; 3 Clinical Science, Philips Healthcare, Chengdu, China

**Keywords:** Magnetic resonance angiography, Deep learning, Coronary artery disease, Coronary CT angiography, Coronary arteries

## Abstract

**Objectives:**

To investigate the feasibility of a deep learning-constrained compressed sensing (DL-CS) method in non-contrast-enhanced modified DIXON (mDIXON) coronary magnetic resonance angiography (MRA) and compare its diagnostic accuracy using coronary CT angiography (CCTA) as a reference standard.

**Methods:**

Ninety-nine participants were prospectively recruited for this study. Thirty healthy subjects (age range: 20–65 years; 50% female) underwent three non-contrast mDIXON-based coronary MRA sequences including DL-CS, CS, and conventional sequences. The three groups were compared based on the scan time, subjective image quality score, signal-to-noise ratio (SNR), and contrast-to-noise ratio (CNR). The remaining 69 patients suspected of coronary artery disease (CAD) (age range: 39–83 years; 51% female) underwent the DL-CS coronary MRA and its diagnostic performance was compared with that of CCTA.

**Results:**

The scan time for the DL-CS and CS sequences was notably shorter than that of the conventional sequence (9.6 ± 3.1 min vs 10.0 ± 3.4 min vs 13.0 ± 4.9 min; *p* < 0.001). The DL-CS sequence obtained the highest image quality score, mean SNR, and CNR compared to CS and conventional methods (all *p* < 0.001). Compared to CCTA, the accuracy, sensitivity, and specificity of DL-CS mDIXON coronary MRA per patient were 84.1%, 92.0%, and 79.5%; those per vessel were 90.3%, 82.6%, and 92.5%; and those per segment were 98.0%, 85.1%, and 98.0%, respectively.

**Conclusion:**

The DL-CS mDIXON coronary MRA provided superior image quality and short scan time for visualizing coronary arteries in healthy individuals and demonstrated high diagnostic value compared to CCTA in CAD patients.

**Critical relevance statement:**

DL-CS resulted in improved image quality with an acceptable scan time, and demonstrated excellent diagnostic performance compared to CCTA, which could be an alternative to enhance the workflow of coronary MRA.

**Key Points:**

- Current coronary MRA techniques are limited by scan time and the need for noise reduction.
- DL-CS reduced the scan time in coronary MR angiography.
- Deep learning achieved the highest image quality among the three methods.
- Deep learning-based coronary MR angiography demonstrated high performance compared to CT angiography.

**Graphical Abstract:**

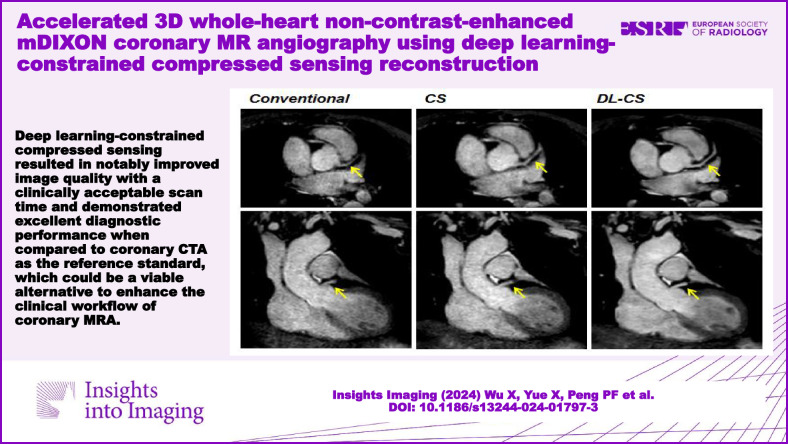

## Introduction

Whole-heart coronary magnetic resonance angiography (MRA) is a noninvasive and radiation-free imaging technique that holds promise for the assessment of coronary arteries [1–3]. MRA has demonstrated high sensitivity, specificity, and negative predictive value for diagnosing coronary artery diseases (CAD) across various clinical scenarios [4]. Currently, the 3D balanced steady-state free precession (bSSFP) sequence is preferred to others for coronary artery visualization [1, 5–8]. However, this imaging technique is sensitive to flow and susceptible to artifacts, which eventually affect image quality [9]. Recent studies performed water–fat separation DIXON-based methods for coronary MRA, revealing substantial enhancement in image quality due to its low susceptibility to field inhomogeneity [10–12]. Additionally, this approach also provides additional available fat information and assists in identifying vascular calcification, luminal stenosis, and plaque burden [13–15]. Furthermore, the modified DIXON (mDIXON) approach provides images with excellent quality and enhanced vessel-lumen-to-fat contrast, and allows an additional signal-to-noise ratio (SNR) boost [9, 16, 17].

Currently, the use of whole-heart mDIXON-based strategies in assessing coronary arteries faces limitations due to the inherent small caliber of coronary arteries, which impedes its routine application in clinical practice [12, 18, 19]. Firstly, the extended scan time may lead to heart rate and respiratory pattern drift, bulk motion, and consequent degradation of the final image quality [12]. Secondly, while higher spatial resolution is highly desirable for coronary MRA, it compromises SNR which is directly proportional to the voxel size [20]. Thus, effective noise removal is essential for high-resolution coronary MRA. There remains an unmet need to reduce the scan time of 3D high-resolution coronary MRA sequences in clinical practice.

Novel approaches such as parallel accelerated techniques and compressed sensing (CS)-based methods enable rapid data acquisition. The use of parallel accelerated techniques, such as sensitivity encoding (SENSE), has led to reduced image acquisition times while maintaining image quality in coronary MRA [21–24]. Despite its promising result, limitations are raised with specific artifacts, including residual aliasing and g-factor noise enhancement, due to the high acceleration factor associated with parallel accelerated techniques [25]. CS-based techniques, which involve fast acquisition through random undersampling of *k*-space combined with iterative image reconstruction, have shown potential in producing higher overall image quality compared to conventional parallel accelerated techniques in coronary MRA [12, 18, 26]. Despite these advancements, selecting optimal sparsity transforms and addressing artifacts remain significant challenges in CS-based techniques, especially with very high acceleration factors leading to the potential reduction in image quality due to insufficient noise removal during reconstruction [27].

Recently, the integration of deep learning (DL) into MR acquisition has demonstrated promising results in enhancing image quality or speeding up the process in coronary MRA [20, 28–32]. However, such a technique, involves training a neural network to identify the most effective transformation from the zero-filled *k*-space to the desired reconstruction. A novel convolutional neural network incorporates multiscale sparsification in a learnable manner to substitute traditional sparsifying transforms and improve the CS approach [33]. The DL-constrained compressed sensing (DL-CS) method, employing the Adaptive-CSNetwork scheme, has shown superior performance in MR reconstructions [28, 34–36]. A useful strategy for achieving a rapid 3D mDIXON coronary MRA would exploit the advantage of CS theory and DL algorithms.

This study aimed to investigate the feasibility of using DL algorithms to enhance image quality in the CS-based non-contrast-enhanced mDIXON coronary MRA. We intended to fully evaluate its performance in both healthy subjects and patients suspected of CAD and hypothesized that the DL-CS technique would expand the scope of clinical application of mDIXON coronary MRA with improved image quality.

## Materials and methods

### Study participants

Ninety-nine participants were prospectively enrolled in this study between October 2021 and January 2023. The study included two subgroups: 30 healthy volunteers (15 females; age range, 20–65 years) with no medical conditions that could interfere with the acquisition and analysis, and 69 patients (35 females; age range, 39–83 years) suspected of CAD scheduled for coronary computed tomography angiography (CCTA). Subjects with pacemakers, defibrillators, or other general contraindications were excluded from the study. No beta-blocker or nitroglycerin was administered to any of the subjects. The institutional review board approved this prospective study, and all study population provided written informed consent.

### MRA protocol

A 3-T MR scanner (Ingenia Elition, Philips Healthcare) equipped with a 16-channel body matrix coil and a 12-channel spine matrix coil was used to perform the coronary MRA examinations. Electrocardiography-triggered and respiratory navigator-gated techniques were used during the entire process of the scan. To identify the relatively quiescent time of the right coronary artery (RCA), one hundred cardiac phase four-chamber images were obtained during the free-breathing of the participants, allowing for the determination of the optimal acquisition window and delay time for the scan. For coronary artery imaging, all healthy volunteers underwent three non-contrast mDIXON coronary MRA scans, including DL-CS, CS, and conventional SENSE approaches. The scan order of the three sequences was randomized for each subject. For patients with suspected CAD, coronary MRA was obtained using the DL-CS sequence. The scanning parameters are presented in Table 1.

**Table 1:** Image parameters

	DL-CS	CS	Conventional
Sequence type	Spoiled gradient echo	Spoiled gradient echo	Spoiled gradient echo
Fat suppression	mDIXON	mDIXON	mDIXON
TR/TE1/TE2 (ms)	4.10/1.32/2.40	4.10/1.32/2.40	4.10/1.32/2.40
Field of view (mm^3^)	265 × 301 × 112	265 × 301 × 112	265 × 301 × 112
Matrix	176 × 201 × 150	176 × 201 × 150	176 × 201 × 150
Acceleration factor	CS 5	CS 5	SENSE 4
Acquisition voxel size (mm^3^)	1.50 × 1.50 × 1.50	1.50 × 1.50 × 1.50	1.50 × 1.50 × 1.50
Reconstruction voxel size (mm^3^)	0.75 × 0.75 × 0.75	0.75 × 0.75 × 0.75	0.75 × 0.75 × 0.75
Bandwidth (Hz/pixel units)	0.36	0.36	0.36
Scale factor	0.6	0.6	0.6
T2 preparation duration (ms)	30	30	30
Acceptance window	5	5	5
Flip angle (degree)	10	10	10

*CS* compressed sensing, *DL* deep learning, *SENSE* sensitivity encoding

### CCTA protocol

All patients also received CCTA utilizing a 256-row CT scanner with a 16 cm wide detector (Revolution CT, GE Healthcare). All examinations were obtained using the prospective ECG-triggered axial acquisition scan mode. Specific scan parameters included a gantry rotation time of 0.28 s, an automatically adjusted tube current range between 400 mA and 800 mA, a tube voltage of 120 kV, and a slice thickness of 0.625 mm using the “stand” reconstruction kernel. Iodinated contrast material (Iomeprol Injection, concentration 400 mgI/mL) was administered to each patient at a traditional weight-dependent rate of 32 mgI/kg/s for a 10-s injection time. The injection flow rate was set by dividing the total contrast volume by the 10-s injection time. Following contrast injection, 30 mL of saline was administered at the same flow rate.

### Image analysis

Each acquisition time was recorded for each volunteer. All original MR images were transferred to the post-processing workstation (IntelliSpace Portal version 9.0.5; Philips Healthcare) for quality assessment.

### Healthy subject study

#### Qualitative image analysis

Four radiologists independently evaluated all the coronary MRA images and blindly to the scan protocol. The quality of coronary artery visualization was assessed using a five-point scale: five for excellent, coronary vessels with clear edges, minimal image noise and no apparent artifacts; four for good, coronary vessels with clear edges, mild image noise and artifacts; three for average, coronary vessels with adequate edges, moderate image noise and artifacts; two for poor, coronary vessels with blurred edges, high image noise, and severe artifacts; one for not visible. An image with a score of 3 or higher was deemed satisfactory and suitable for diagnosis. The original image quality scores from the four readers were utilized to assess consistency, while the final score for each participant was determined through negotiation among the four readers and was utilized for comparison between the three sequences.

#### Quantitative objective assessment

SNR and contrast-to-noise ratio (CNR) of the original image were calculated to compare the three techniques. For the measurement of blood intensity in the coronary arteries, the region of interest (ROI) was carefully placed in the lumen of the proximal areas of the LAD, left circumflex (LCX), and RCA, with the ROI size set as large as possible (2–5 mm) to mitigate partial volume effects. The myocardium adjacent to each coronary artery was selected to measure the myocardial signal intensity (SI). The noise was determined using the mean standard deviation (SD) of pixel values from two large air ROIs selected outside of the body. Similar ROIs were used in all image sets from the three sequences for each patient unless artery motion was detected. The following equations were used to calculate SNR and CNR [5]:

$${{{\rm{SNR}}}}={{{\rm{Mean}}}}\left({{{{\rm{SI}}}}}_{{{{\rm{coronary\; artery}}}}}\right)/{{{{\rm{SD}}}}}_{{{{\rm{air}}}}}$$

$${{{\rm{CNR}}}}=\left({{{{\rm{SI}}}}}_{{{{\rm{coronary\; artery}}}}}-{{{{\rm{SI}}}}}_{{{{\rm{myocardium}}}}}\right)/{{{{\rm{SD}}}}}_{{{{\rm{air}}}}}$$

### Patient study

An observer evaluated the diagnostic performance of DL-CS mDIXON coronary MRA, who was blinded to participants’ clinical information and the results of CCTA. The luminal diameter reduction of ≥ 50% in a reference diameter ≥ 1.5 mm was defined as significant coronary vessel stenosis [37]. Two independent observers analyzed significant stenosis on CCTA images with CCTA serving as the non-invasive clinical reference standard. Consensus was reached through negotiation in case of disagreement. The number of stenoses on coronary MRA and its diagnostic performance for detecting significant stenosis, including sensitivity, specificity, positive predictive value (PPV), negative predictive value (NPV), and accuracy on a per-patient, per-vessel, and per-segment basis, were evaluated in comparison to CCTA.

### Statistical analysis

Statistical analysis was conducted using the SPSS statistical software (version 25.0) and MedCalc software (version 19.3). All continuous variables were presented as mean ± SD. Friedman’s test was employed to assess the mean scan time, visual evaluation scores, SNR, and CNR of the three sequences. The weighted Kappa test was utilized to evaluate the inter-observer agreement (kappa value > 0.7 was considered excellent, 0.4–0.7 was considered good, and < 0.4 was considered bad) [38]. Bland–Altman analysis was used to visually compare the difference between paired measures against their averages. A *p*-value of less than 0.05 indicated a statistical difference.

## Results

The based characteristics for all participants are shown in Tables 2 and  3. All coronary MRA acquisitions were successfully performed for all healthy volunteers and patients suspected of CAD.

**Table 2:** The characteristics of the healthy subjects (*n* = 30)

Characteristics	Results
Age (y)	28 ± 11
Age range (y)	20–65
Sex (female/male)	15/15
Height (cm)	166 ± 9
Weight (kg)	61 ± 14
Body mass index (kg/m^2^)	21.7 ± 3.1
Systolic blood pressure (mmHg)	116 ± 14
Diastolic blood pressure (mmHg)	77 ± 10
Heart rate (beats/min)	74.0 ± 8.7

**Table 3:** Baseline characteristics for the patient study

Characteristics	Results
Age (years)	59 ± 10
Sex (female/male)	35/34
Body mass index (kg/m^2^)	24.0 ± 3.1
Hemodynamic data	
Resting SBP (mmHg)	135 ± 20
Resting DBP (mmHg)	86 ± 9
Heart rate (beats/min)	74 ± 11
Cardiovascular risk factors	
Hypertension, *n* (%)	28 (40.6)
Hyperlipidemia, *n* (%)	28 (40.6)
Diabetes mellitus, *n* (%)	9 (13)
Prior myocardial infarction, *n* (%)	4 (5.8)
Smoking, *n* (%)	24 (34.8)
Family history of CAD, *n* (%)	12 (17.4)
Significant disease prevalence of CAD, *n* (%)	25 (36.2)
1 Coronary vessel disease, *n* (%)	13 (18.8)
2 Coronary vessel disease, *n* (%)	4 (5.8)
3 Coronary vessel disease, *n* (%)	8 (11.6)
CAC score prevalence	
0, *n* (%)	26 (37.7)
1–100, *n* (%)	15 (21.7)
101–300, *n* (%)	12 (17.4)
> 300, *n* (%)	16 (23.2)
CAD-RADS score	
0–2 (< 50% stenosis), *n* (%)	44 (63.8)
3 (50–69% stenosis), *n* (%)	12 (17.4)
4 (70–99% stenosis), *n* (%)	13 (18.8)

*CAC* coronary artery calcium, *CAD* coronary artery disease, *CAD-RADS* coronary artery disease-reporting and data system, *DBP* diastolic blood pressure, *SBP* systolic blood pressure

### Healthy subject study

For healthy subjects, the acquisition time of the DL-CS and CS coronary MRA was shorter compared to the conventional method (9.6 ± 3.1 min vs 10.0 ± 3.4 min vs 13.0 ± 4.9 min; *p* < 0.001), with no significant difference between DL-CS and CS methods (*p* > 0.05).

#### Qualitative image analysis

Regarding the subjective evaluation of image quality, the DL-CS group achieved the highest quality scores than the conventional and CS coronary MRA (4.10 ± 0.96 vs 3.33 ± 0.99 vs 3.27 ± 1.01, *p* < 0.001). However, no significant difference in quality score was observed between the conventional and CS approaches (*p* > 0.05). The comparison of subjective image quality among the three sequences is shown in Supplementary Table 1. The weighted kappa value for inter-observer agreement for image quality score was excellent (kappa = 0.75–0.97).

#### Quantitative objective assessment

**Fig. 1:**
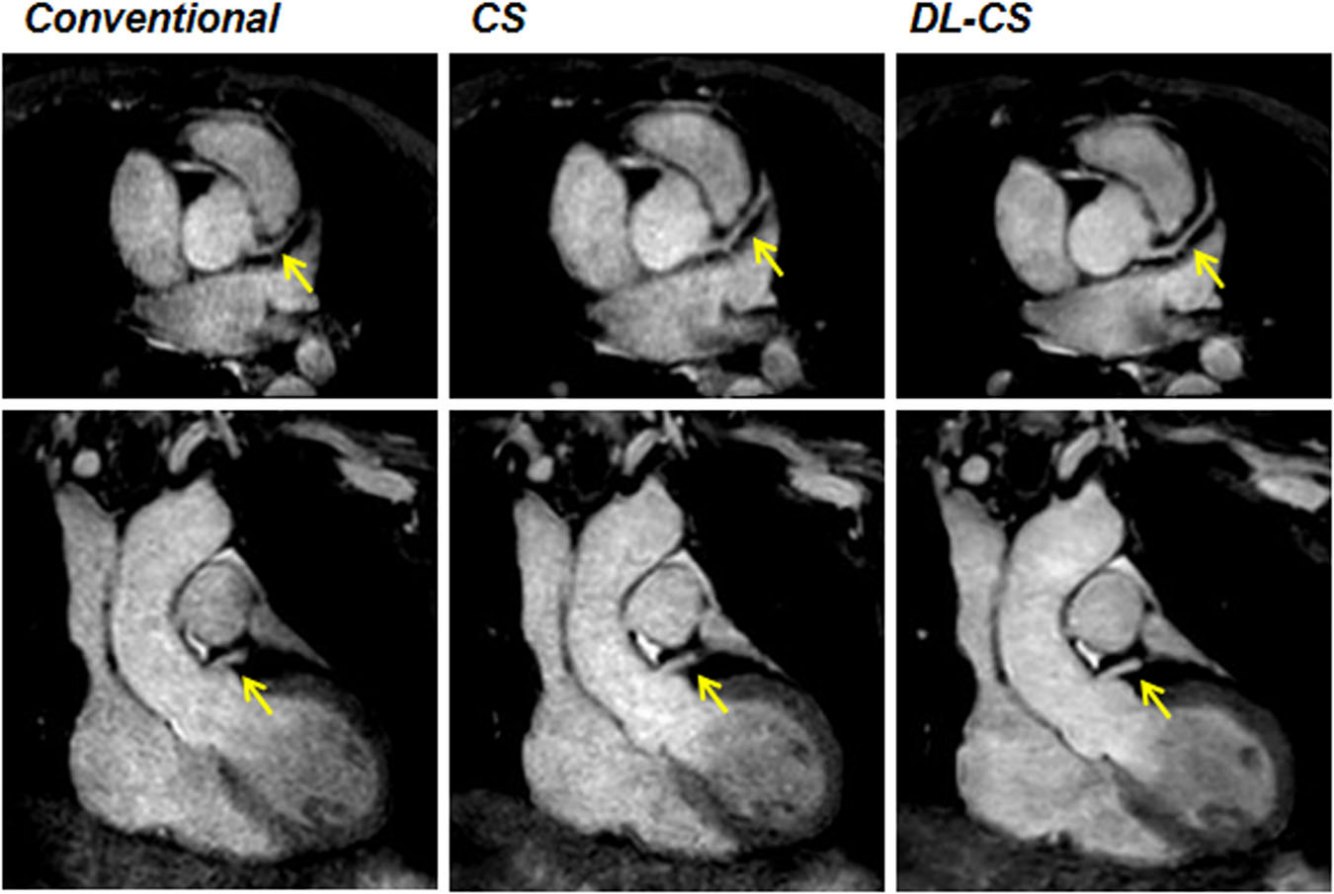
A 49-year-old female volunteer. Comparison of image qualities among the three mDIXON sequences in visualizing the origins and proximal courses of the left coronary artery. CS, compressed sensing; DL, deep learning

**Fig. 2:**
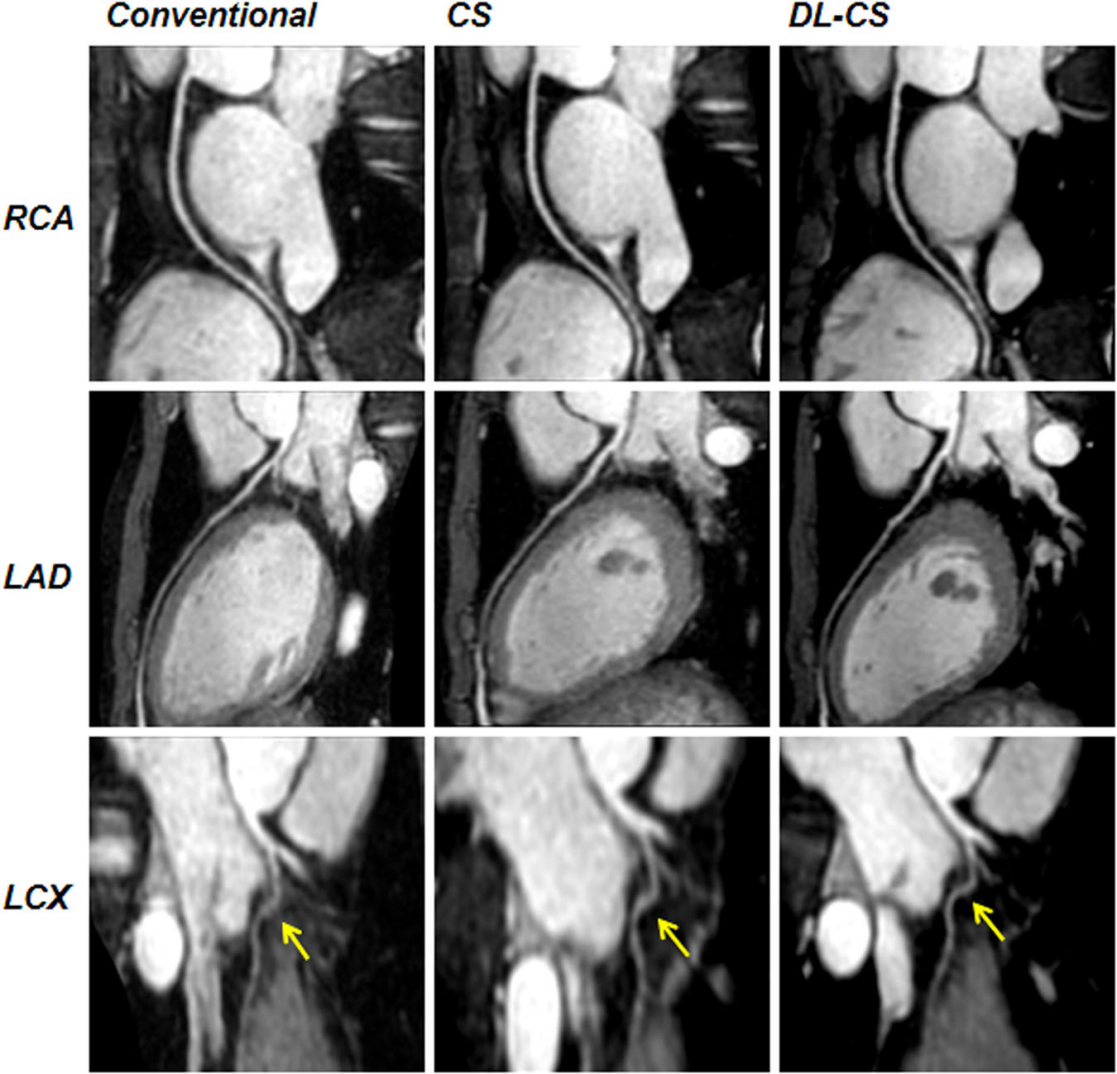
The curved planar reformation images were obtained from a healthy 21-year-old male participant using conventional, CS, and DL-CS mDIXON coronary MRA sequences. The image quality of the RCA, LAD artery, and left circumflex on the DL-CS image was superior to the conventional and CS methods, especially on the LCX visualization (yellow arrows). CS, compressed sensing; DL, deep learning; LAD, left anterior descending artery; LCX, left circumflex; RCA, right coronary artery

**Fig. 3:**
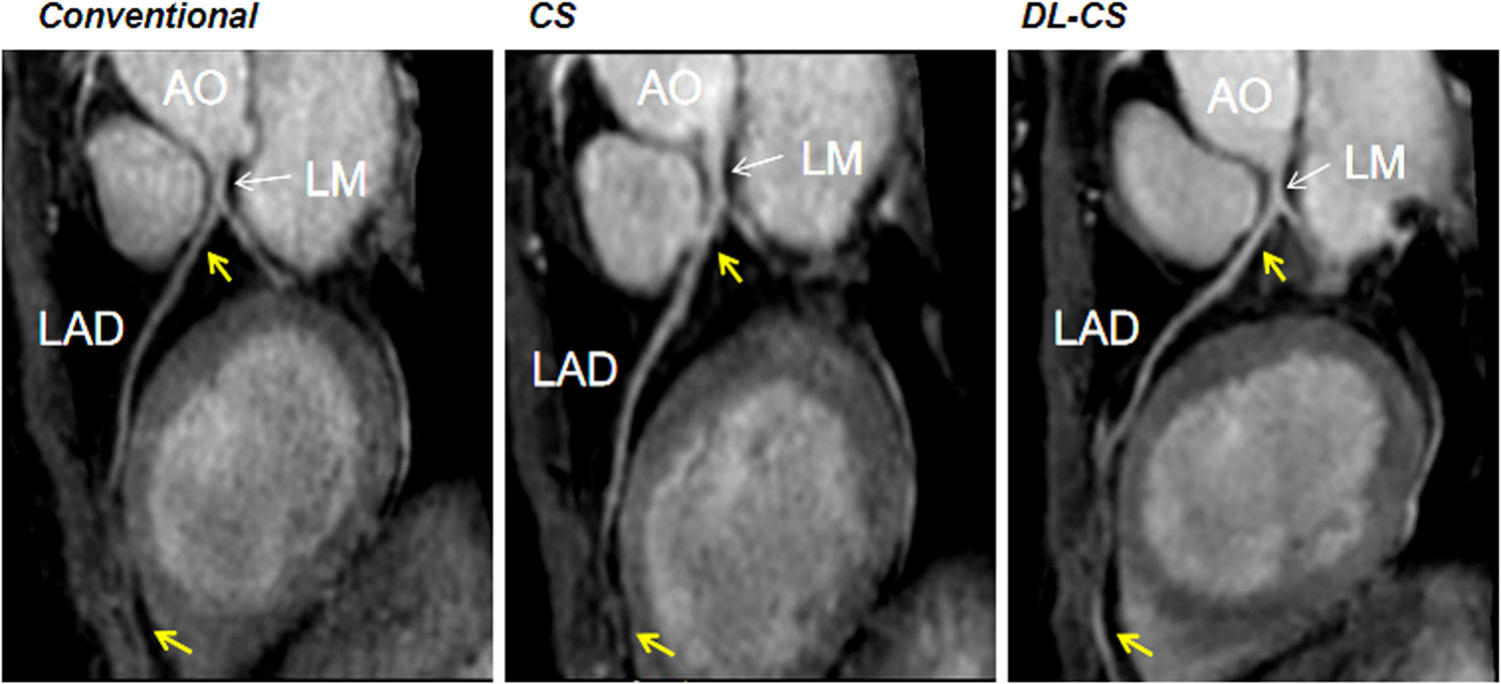
Curved planar reformation images of the LAD from three coronary MRA approaches were obtained from a healthy 49-year-old female participant. The proximal and distal segments in the conventional and CS images exhibited less clarity compared to the DL-CS image (yellow arrows). AO, aorta; CS, compressed sensing; DL, deep learning; LAD, left anterior descending artery; LM, left main artery

**Fig. 4:**
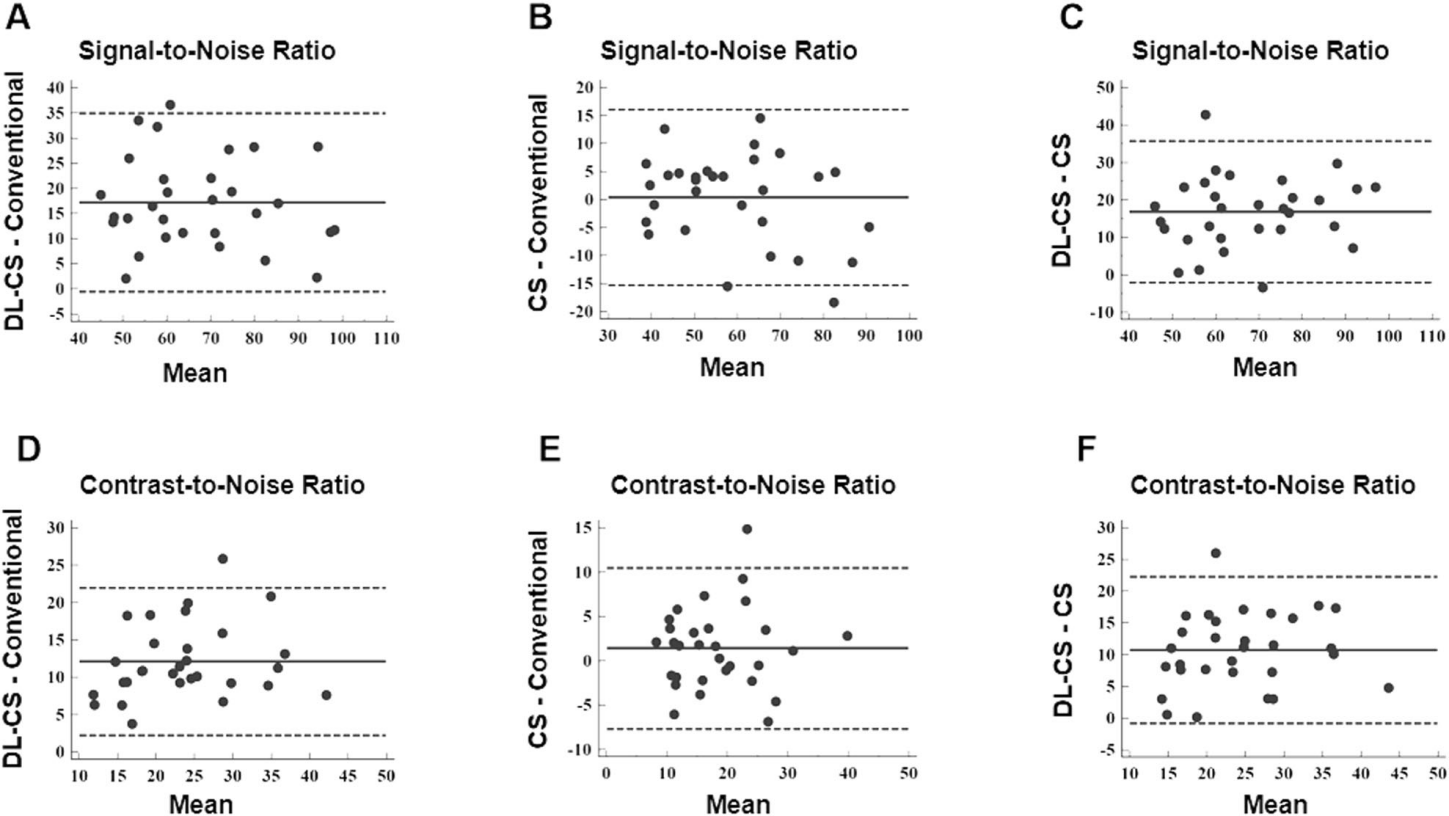
Bland–Altman analysis for SNR and CNR for DL-CS—conventional (**A**, **D**), CS—conventional (**B**, **E**), and DL-CS—CS (**C**, **F**). The *y*-axis provided the difference between the paired measures, while the *x*-axis provided the mean of the paired measures. The solid and dotted lines provided the mean difference and the 95% limits of agreement. CS, compressed sensing; DL, deep learning

Among 30 healthy participants, 440 coronary segments with a diameter ≥ 1.5 mm were identified, with 438 (99.5%) for the DL-CS, 429 (97.5%) for the CS, and 426 (96.8%) for the conventional method available for evaluation. The SNRs and CNRs are summarized in Table 4. The SNRs and CNRs of DL-CS were highest among the three techniques (*p* < 0.001), but those were no significant differences in the conventional and CS coronary MRA approaches (*p* > 0.05). The comparison of image qualities among three groups in showing the origins and proximal courses of the left coronary artery is depicted in Fig. 1 and representative CPR images of three methods from two healthy volunteers are shown in Figs. 2 and  3, where the DL-CS showed superior image quality with fewer blurring artifacts. The Bland–Altman analysis indicated acceptable agreement for the DL-CS—conventional, CS—conventional, and DL-CS—CS pairs concerning their corresponding SNRs and CNRs (Fig. 4).

**Table 4:** Comparison of SNRs and CNRs from three methods with mDIXON coronary MRA

	Conventional	CS	DL-CS
Mean SNR	58.88 ± 16.98	59.24 ± 14.71	76.06 ± 15.93^a,b^
RCA	62.42 ± 20.38	61.73 ± 16.50	79.65 ± 18.77^a,b^
LAD	56.19 ± 14.89	57.48 ± 17.08	73.40 ± 17.07^a,b^
LCX	58.02 ± 20.92	58.51 ± 15.77	75.12 ± 17.88^a,b^
Mean CNR	17.61 ± 7.71	19.00 ± 7.87	29.70 ± 8.67^a,b^
RCA	21.10 ± 11.31	22.36 ± 10.34	34.61 ± 12.95^a,b^
LAD	15.85 ± 10.13	16.91 ± 10.90	26.02 ± 11.38^a,b^
LCX	15.88 ± 11.51	17.73 ± 9.68	28.48 ± 11.37^a,b^

*CNR* contrast-to-noise ratio, *CS* compressed sensing, *DL* deep learning, *LAD* left anterior descending artery, *LCX* left circumflex, *RCA* right coronary artery, *SNR* signal-to-noise ratio

^a^ DL-CS vs conventional, *p* < 0.001

^b^ DL-CS vs CS, *p* < 0.001

### Patient study

Twenty-five out of the sixty-nine patients presented with significant CAD on CCTA. Cardiovascular risk factors, and significant disease prevalence of CAD are shown in Table 3. The acquisition time interval between the coronary MRA and the CCTA ranged from 0 day to 15 days, with an average of 2 days.

**Fig. 5:**
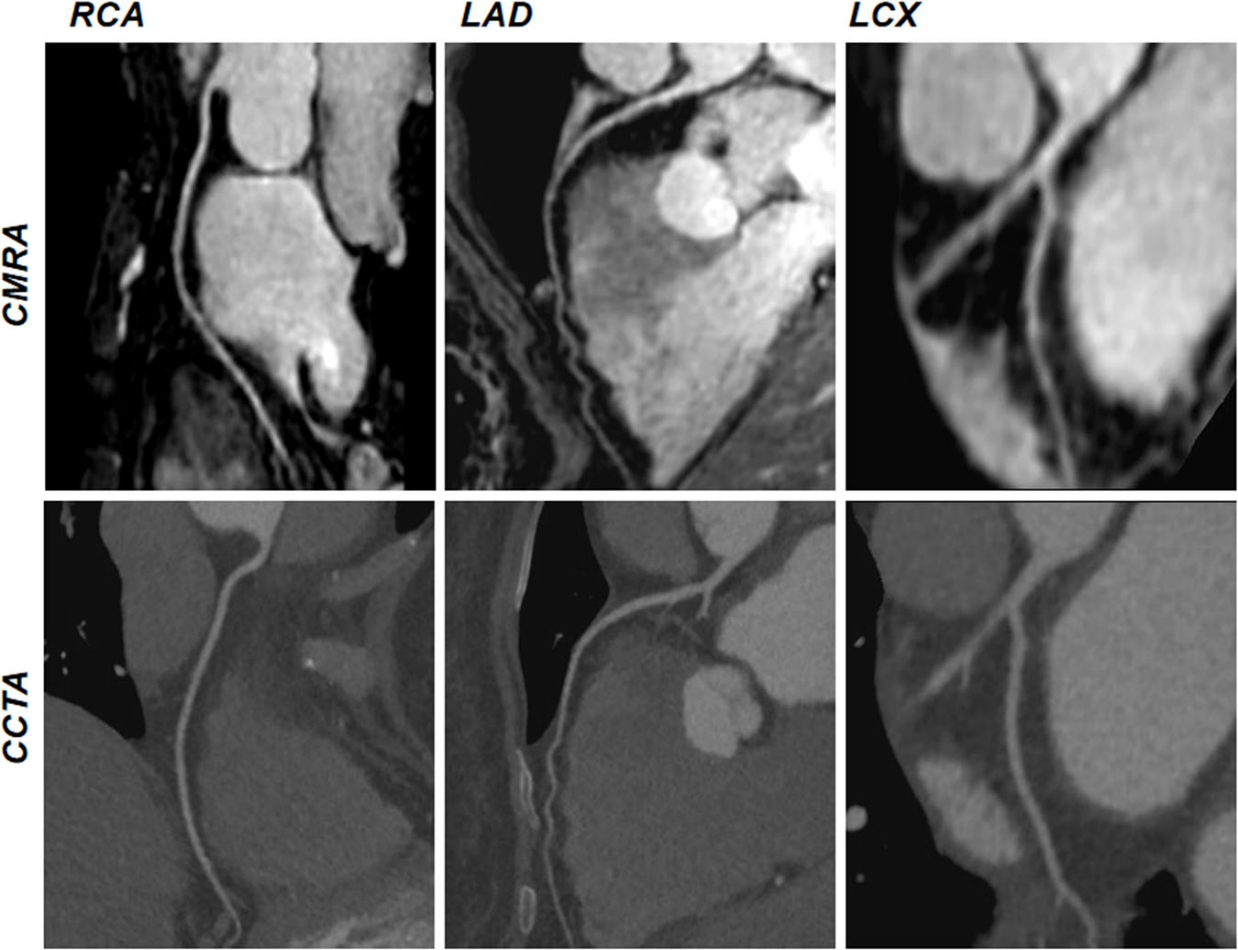
Non-contrast DL-CS mDIXON coronary MRA images of a 60-year-old female patient with normal coronary arteries. coronary MRA images are depicted in the top row, displaying the right coronary artery, LAD artery, and left circumflex. The corresponding reformatted images obtained with contrast-enhanced CCTA are shown in the bottom row. CMRA, coronary magnetic resonance angiography; CCTA, coronary computed tomography angiography

**Fig. 6:**
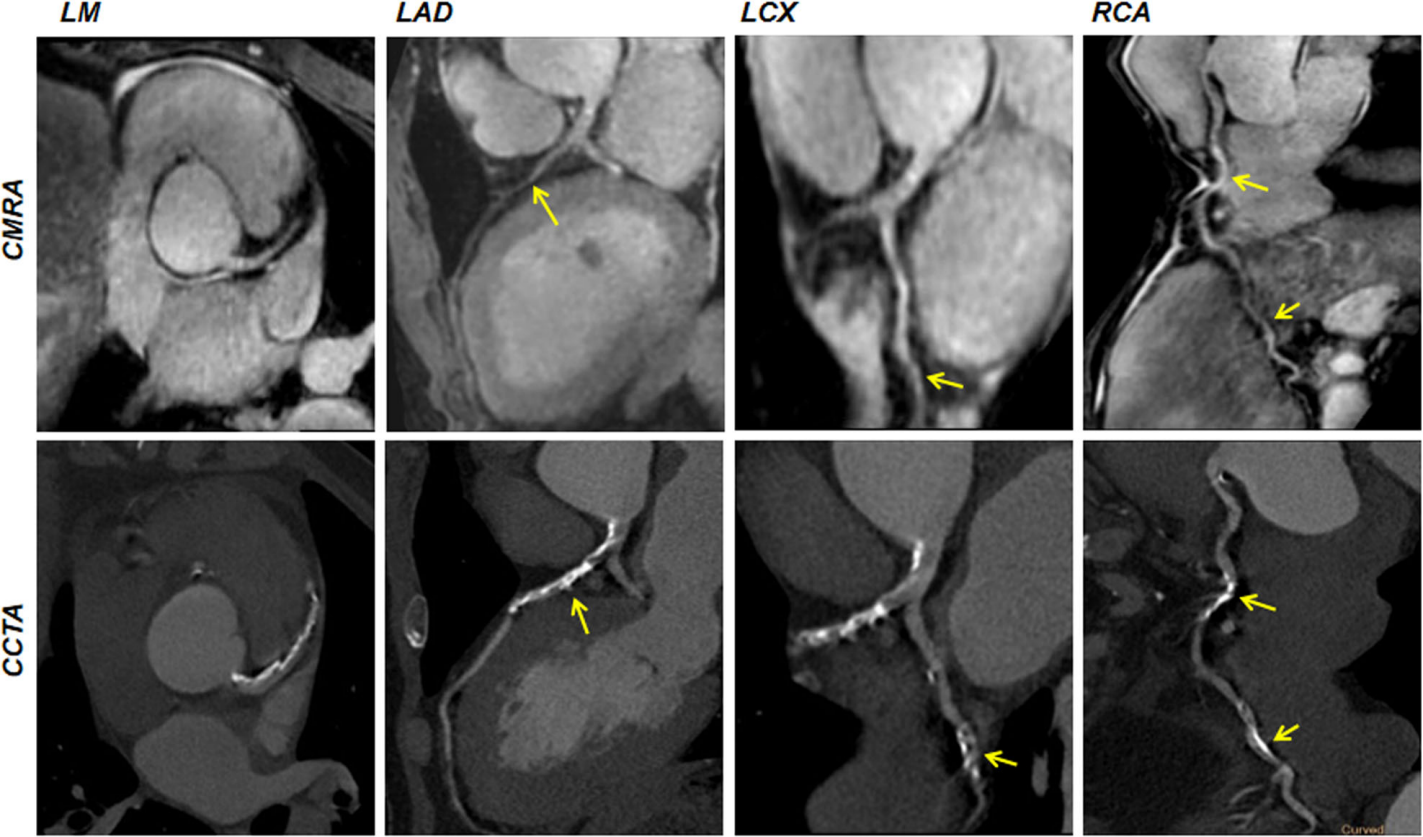
Curved planar reformation coronary MRA and CCTA images from a 63-year-old male patient with diffuse calcification and significant stenosis (≥ 50% diameter stenosis) (yellow arrows). Coronary MRA images show stenosis matching the lesion detected by CCTA. CCTA, coronary computed tomography angiography; CMRA, coronary magnetic resonance angiography; LAD, left anterior descending artery; LCX, left circumflex; LM, left main artery; RCA, right coronary artery

Among the 69 patients, CCTA images revealed 930 segments with a reference luminal diameter ≥ 1.5 mm, out of which 885 segments (95.2%) in the coronary MRA were deemed diagnostic. The per-patient sensitivity, specificity, PPV, NPV, and accuracy of DL-CS coronary MRA were 92.0%, 79.5%, 71.9%, 94.6%, and 84.1%, respectively, those per vessel were 82.6%, 92.5%, 76.0%, 94.9%, and 90.3%, respectively, and those per segment were 85.1%, 98.0%, 79.7%, 98.6%, and 96.9%, respectively (Table 5). Representative coronary MRA and CCTA images of patients are depicted in Figs. 5 and 6.

**Table 5:** Diagnostic performance of DL-CS mDIXON coronary MRA in detecting significant CAD as defined by CCTA

	Accuracy (%)	Sensitivity (%)	Specificity (%)	PPV (%)	NPV (%)
Per patient	84.1 (58/69)	92.0 (23/25)	79.5 (35/44)	71.9 (23/32)	94.6 (35/37)
Per vessel	90.3 (187/207)	82.6 (38/46)	92.5 (149/161)	76.0 (38/50)	94.9 (149/157)
RCA	91.3 (63/69)	81.8 (9/11)	93.1 (54/58)	69.2 (9/13)	96.4 (54/56)
LAD	88.4 (61/69)	80.0 (20/25)	93.2 (41/44)	87.0 (20/23)	89.1 (41/46)
LCX	91.3 (63/69)	90.0 (9/10)	91.5 (54/59)	64.3 (9/14)	98.2 (54/55)
Per segment	96.9 (857/884)	85.1 (63/74)	98.0 (794/810)	79.7 (63/79)	98.6 (794/805)

*LAD* left anterior descending, *LCX* left circumflex, *NPV* negative predictive value, *PPV* positive predictive value, *RCA* right coronary artery

## Discussion

This study assessed a DL-CS approach with 3D whole-heart non-contrast-enhanced mDIXON coronary MRA in both healthy subjects and patients suspected of CAD. The preliminary results revealed that the DL-CS sequence could provide higher SNR and CNR, along with favorable image quality for visualizing coronary arteries in a short acquisition time compared to conventional and CS approaches in healthy subjects. Furthermore, it exhibited excellent diagnostic performance in patients, with CCTA serving as the reference standard.

DIXON-based strategies showed promising results in coronary MRA because of their unique advantages [10–12]. Given that coronary arteries are surrounded by epicardial fat, inadequate fat suppression can compromise vessel delineation [10]. Previous studies have indicated that the DIXON-based method, when compared to conventional fat suppression, improved coronary MRA image quality at both 1.5 T [39] and 3.0 T [10]. Additionally, compared to bSSFP, the DIXON-based technique displayed reduced sensitivity to magnetic field inhomogeneities [17, 40], resulting in fewer artifacts and enhanced vessel wall sharpness at distal anatomical borders [9]. Moreover, the presence of residual high-intensity fat signals can lead to motion-induced ghosting artifacts, negatively impacting image quality [41]. Leveraging the phase shifts caused by water–fat resonance frequency difference, the Dixon-based technique can separate water from fat, potentially reducing susceptibility to motion artifacts in the water images [10].

The DL-CS method demonstrated promising potential for rapid mDIXON coronary MRA. The major challenge in whole-heart coronary MRA has been the extended scan time, leading to motion artifacts. Parallel accelerated techniques [21–23] and CS-based accelerated methods [18, 26] could be used for fast data acquisition. Previous studies have shown that CS-based coronary MRA can reduce the scan time with favorable image quality compared to conventional parallel accelerated approaches [12, 18, 42]. Recently, DL reconstruction has been proposed for de-noising in coronary MRA [3]. Leveraging the “learning noise” strategy, DL can effectively optimize the de-noising level and preserve a good edge, resulting in improved image quality [3]. High image quality was obtained using the DL-based reconstruction, which indicated that the DL method reduced image noise to preserve image quality while obtaining high spatial resolution [20, 43]. Yokota et al [20] evaluated the effects of DL reconstruction on image quality of high-resolution coronary MRA with ten healthy volunteers at 3 T. Additionally, Qi et al [29] and Fuin et al [30] developed DL techniques for bSSFP coronary MRA on a 1.5-T MR scanner. Hosseini et al [31] proposed the use of self-consistent robust artificial neural networks for *k*-space interpolation to enhance coronary MRA reconstruction quality. In contrast, our study combined CS and DL to achieve rapid 3D mDIXON coronary MRA, which exploited the advantage based on CS theory and DL algorithm. The DL-CS approach significantly reduced acquisition time compared to conventional methods, while also improving SNR, CNR, and image quality when compared to CS and conventional methods in healthy subjects. Furthermore, DL-CS coronary MRA successfully demonstrated excellent diagnostic performance in 69 patients suspected of CAD, using CCTA as the reference standard. Especially, for patients with pregnancy or hypersensitivity to iodinated contrast agents, non-contrast-enhanced coronary MRA could be an alternative modality for CCTA.

Several additional critical aspects of the mDIXON coronary MRA were considered in this study. To mitigate bias, the DL-CS, CS, and conventional sequences were scanned in random order. As the scan time increases, so does the patient’s fatigue and movement, as well as changes in respiration condition and heart rate, ultimately reducing acquisition efficiency and compromising image quality. Additionally, we employed one hundred cardiac phase images to identify a relatively quiescent period for imaging coronary arteries, recognizing the significant impact of heart rate on image quality. For patients with a high heart rate, the acquisition window was generally set at end-systole to obtain good image quality, consistent with findings by Wu et al [44]. Certainly, pre-scan breathing training and instructions to maintain calm breathing during the scan are also critical for obtaining high-quality coronary artery images.

Several limitations were present in this study. The image quality did not allow for a completely blinded evaluation, as DL-CS coronary images were easily distinguishable from conventional and CS coronary images due to reduced noise and superior image quality. For healthy participants, there was no reference standard (e.g., CCTA) available for comparing coronary artery image quality among the three groups. Furthermore, it is important to note that the patients included in this study did not undergo invasive coronary X-ray angiography as the “gold standard” and CCTA may not provide a definite diagnosis of obstructive CAD in some cases. Lastly, a limited number of healthy individuals and patients were enrolled in this study, which sufficed for demonstrating the technical feasibility. While we did not conduct studies on vessel wall and plaque characterization, we anticipate that future technical advancements in MRI will facilitate the analysis of vessel wall and plaque.

In conclusion, the potential of 3D whole-heart non-contrast-enhanced DL-CS mDIXON coronary MRA has been confirmed in both healthy subjects and individuals with suspected CAD. The proposed DL-CS method notably enhanced image quality with a clinically acceptable scan duration and demonstrated excellent diagnostic performance when compared to CCTA as the reference standard, which could be a viable alternative to enhance the clinical workflow of coronary MRA.

## Supplementary information

Electronic supplementary material

## Data Availability

The data used during the current study are available from the corresponding author upon reasonable request.

## Abbreviations

bSSFP: Balanced steady-state free precession
CAD: Coronary artery disease
CCTA: Coronary computed tomography angiography
CS: Compressed sensing
DL: Deep learning
LAD: Left anterior descending
LCX: Left circumfex
mDIXON: Modified DIXON
MRA: Magnetic resonance angiography
RCA: Right coronary artery
SENSE: Sensitivity encoding
