# Supplementary material for: Accelerated 3D whole-heart non-contrast-enhanced mDIXON coronary MR angiography using deep learning-constrained compressed sensing reconstruction
Source: Insights Imaging. 2024 Sep 19;15:224. doi: 10.1186/s13244-024-01797-3 (PMC11413263; doi:10.1186/s13244-024-01797-3)
Supplement: Supplementary file 1 — Electronic supplementary material [file 13244_2024_1797_MOESM1_ESM.pdf]

# Accelerated 3D whole-heart non-contrast-enhanced mDIXON coronary MR angiography using deep learning-constrained compressed sensing reconstruction

# ELECTRONIC SUPPLEMENTARY MATERIAL

**Supplementary Table 1.** Subjective image quality (distribution) comparison among the three sequences.

| Quality score | DL-CS | | | | CS | | | | Conventional | | | |
|---|---|---|---|---|---|---|---|---|---|---|---|---|
| | Reader 1 | Reader 2 | Reader 3 | Reader 4 | Reader 1 | Reader 2 | Reader 3 | Reader 4 | Reader 1 | Reader 2 | Reader 3 | Reader 4 |
| 1 | 0 | 0 | 0 | 0 | 0 | 0 | 0 | 0 | 0 | 0 | 0 | 0 |
| 2 | 2 | 2 | 2 | 1 | 6 | 7 | 5 | 5 | 7 | 7 | 6 | 7 |
| 3 | 6 | 5 | 5 | 7 | 13 | 12 | 14 | 14 | 13 | 12 | 13 | 11 |
| 4 | 9 | 12 | 12 | 10 | 6 | 7 | 6 | 7 | 5 | 7 | 7 | 9 |
| 5 | 13 | 11 | 11 | 12 | 5 | 4 | 5 | 4 | 5 | 4 | 4 | 3 |
| Kappa value | 0.75-0.93 | | | | 0.80-0.97 | | | | 0.77-0.90 | | | |
